# Does Long-Term Care Substitute for Hospital Care? Evidence From the China Health and Retirement Longitudinal Survey

**DOI:** 10.1093/geroni/igaa057.275

**Published:** 2020-12-16

**Authors:** Wei Yang

**Affiliations:** King’s College London, London, United Kingdom

## Abstract

Publicly funded long-term care (LTC) support is shrinking in many countries despite continuing increases in the number of older people who need care. Evidence has shown that the LTC services have an impact on the efficient use of the resources in the health care sector by reducing rates of admission and associated costs through assisting older people with daily living. This paper seeks to examine whether and to what extent these services are substitutes. We use a fixed-effect instrumental variable GMM model to predict the effect of long-term care services on the utilisation of outpatient and inpatient care services. Data are drawn from China Health and Retirement Longitudinal Survey 2011, 2013 and 2015. Our findings suggest that LTC significantly reduces the use of outpatient care but not inpatient care. We have also found LTC use is concentrated among the rich, but the substitution effects are stronger among the poor compared to the rich. This indicates that the poor would benefit more from subsided LTC services. We urge the Chinese government to take action to develop its formal LTC system and to channel more resources to its LTC system, which will benefit the older population in general, and the poor in particular.

